# Comparison of DNA histograms by standard flow cytometry and image cytometry on sections in Barrett's adenocarcinoma

**DOI:** 10.1186/1472-6890-8-5

**Published:** 2008-05-30

**Authors:** Qin Huang, Chenggong Yu, Xiaoqi Zhang, Raj K Goyal

**Affiliations:** 1 Departments of Pathology, VA Boston Healthcare system and Providence VA Medical Center and Brown Medical School, Providence, RI 02901, USA; 2 Department of Internal Medicine, VA Boston Health Care System and Harvard Medical School, 1400 VFW Parkway, West Roxbury, MA 02132, USA

## Abstract

**Background:**

The purpose of this study was to compare DNA histograms obtained by standard flow cytometry (FC) and high fidelity image cytometry on sections (ICS) in normal gastrointestinal mucosa and Barrett's adenocarcinoma (BAC).

**Methods**

Archival formalin-fixed paraffin-embedded tissue blocks of 10 normal controls from 10 subjects and 42 BAC tissues from 17 patients were examined. DNA FC was performed using standard techniques and ICS was carried out by Automated Cellular Imaging System (ACIS). DNA ploidy histograms were classified into diploid with peak DNA index (DI) at 0.9–1.1, and aneuploid with peak DI > 1.1. DI values of aneuploid peaks were determined. Additionally, for DNA ICS, heterogeneity index (HI) representing DNA content heterogeneity, and histograms containing cells with DI > G2 were also identified.

**Results**

All control samples were diploid by both FC and ICS analyses. In BAC, FC showed diploid peaks in 29%, diploid peaks with additional aneuploid or tetraploid peaks in 57%, and 14% of the samples, respectively. In contrast, ICS showed aneuploid peaks in all the cases with peak DI > 1.25; 37 cases had peak DI between 1.25 and 2.25; and 5 cases had peak DI > 2.25. HI values (mean ± SD) were 11.3 ± 1.1 in controls and 32.4 ± 8.5 in BAC (p < 0.05). Controls had no G2 exceeding cells. However, 19/37 (51%) of the cases with primary peak DI < 2.25 had cells exceeding 9N.

**Conclusion:**

ICS detects DNA aneuploidy in all BAC samples while FC missed the diagnosis of aneuploidy in 29%. In addition, ICS provides more information on HI and G2 exceeding rates.

## Background

DNA ploidy determination has been proposed to be useful in discriminating between benign and malignant lesions, identifying patients at high risk for developing dysplasia or carcinoma, monitoring neoplastic progression, and predicting outcomes and responses to treatment of cancer [1,2]. However, DNA ploidy determination in gastrointestinal solid tumors has not achieved general clinical use, in part, due to the lack of reliable diagnosis of aneuploidy [3].

DNA ploidy is determined by generating a DNA histogram that depicts frequency distribution with different DNA contents of various cells. To generate DNA histograms, standard flow cytometry (FC) has been used frequently in neoplastic conditions including Barrett's esophagus associated neoplastic lesions. It has been suggested that baseline DNA content abnormalities as determined by FC are important predictors of progression to Barrett's adenocarcinoma (BAC) [4,5]. However, reliability of FC in diagnosing aneuploidy is questionable. For example, by standard FC, up to one third of BAC are reported to be diploid and negative for aneuploidy [6-8]. If true, this finding would suggest that almost one third of pre-cancer neoplastic lesions may progress to BAC without showing aneuploidy. However, it is unknown whether the negative diagnosis of aneuploidy in BAC represents a true diploid status or a false negative result related to the technique of FC.

Two main types of errors that influence FC DNA ploidy results are: 1) technical errors related to measurement of DNA content of individual cells; and 2) sampling errors due to inadequate selection of cancer cells in the study sample. A large number of technical improvements in cell separation and analytical techniques, over the years, have improved the technical aspects of DNA content determination of single cells as well as analysis and interpretation of the DNA histograms [4]. However, poor sampling of cancer cells continues to be a major limitation of FC ploidy analysis, because cell suspension of cancerous tissue used for standard FC is a mixture of cancer cells with an unknown proportion of diploid non-epithelial, non-cancer cells. Shankey [9] has pointed out that in order not to overlook aneuploidy, it is necessary to document that at least 20% of cells in the sample are cancer cells. However, in standard FC, proportion of cancer and non-cancer cells in the sample remains unknown. A comparison of standard FC and double labeled FC, using samples enriched with cancer cells, showed that many BAC that were diploid by standard FC were found to be aneuploid by the double-labeled FC [10]. This observation suggests that the high rate false negative aneuploidy by standard FC may be due to poor selection of cancer cells in the sample.

In order to avoid sampling errors, DNA ploidy analysis has been performed by image cytometry on tissue imprints, cell suspensions ('cytospin' preparations) or directly on tissue sections [11]. Imprint preparations require fresh tissue and this technique favors selection of abnormal, loosely distributed epithelial cells and lymphocytes [11]. High fidelity DNA histograms on single dispersed cells by image cytometry provide better identification of minor clones [12-14]. Studies on breast cancer have shown that in addition to DNA aneuploidy, increased cellular DNA content heterogeneity and elevated 5N or 9N exceeding fraction (depending upon DI of the G0/G1 peak) may represent 'unstable aneuploidy' that may identify severe genomic and chromosomal instability and progressive neoplastic lesions and provide additional prognostic indicators [13-15]

Image cytometry on tissue sections (ICS) is designed to study the microscopically defined cell population; only cancer cells are included for analysis of DNA ploidy status. DNA histograms by ICS have also been improved with better instrumentation and software that can also detect rare events with high fidelity [16-20]. ICS is performed on fixed tissue sections, whereas DNA FC has usually been performed on fresh tissue. However, FC and image cytometry on dispersed cells can also be performed on fixed tissues and studies have shown that use of either fresh or archival formalin-fixed paraffin-embedded tissues with different protocols show generally similar DNA histograms [9,11,21]. The advantage of using formalin fixed tissue is the convenience in clinical practice and the possibility for studies in archival tissues.

The purpose of the present study was to compare DNA histograms obtained by standard FC and by ICS using the Automated Cellular Imaging System (ACIS) [18,20]on archived, formalin-fixed BAC tissues.

## Methods

The study protocol was approved by the Institutional Review Boards of the Veterans Affairs (VA) Boston Healthcare System and the Providence VA Medical Center.

### Tissues

From the database of VA Boston Health Care System and Providence VA Medical Center, we randomly selected 17 patients with BAC who underwent surgical resection between 1991 through 2005. The archival formalin-fixed paraffin-embedded tissue blocks were retrieved and 42 tumor-containing blocks (1–3 different blocks per patient) were included in this study. More than one tumor-containing block was available in 12 patients. Results from these blocks were also used to assess the reproducibility of the diagnosis of DNA ploidy in different regions of the same tumor. Another 10 archival surgical resection tissue blocks from 10 non-tumor patients, including 5 normal stomachs and 5 normal colons, were used as controls.

### Histological evaluation

For histological confirmation, a 5 μm-thick section was cut, hematoxylin and eosin (H&E) stained, and evaluated by an experienced gastrointestinal pathologist (Q.H.). The diagnosis of BAC was made when individual malignant cells or abortive glands were seen to invade through the basement membrane into the laminar propria, muscularis mucosa and/or beyond [22].

### Flow cytometry (FC)

Standard FC was performed according to a conventional protocol and the manufacturer's instruction. Briefly, two 50 μm thick sections adjacent to the 5 μm-thick section, used for histological diagnosis, were cut from tissue blocks. Tissue sections were de-paraffinized in xylene, re-hydrated through graded ethanol to distilled water, and allowed to hydrate overnight at room temperature. The tissue was then minced mechanically, filtered through a 70 μm nylon mesh, and incubated for 90 minutes at 37°C in the 0.5% pepsin solution (Sigma Chemical Co., St. Louis, MO, USA) at pH 1.5 with intermittent vortexing. After incubation, the centrifuged cell pellet was re-suspended in phosphate buffered saline (PBS) with 1% bovine serum albumin (BSA), and filtered through a 53 μm mesh. After centrifugation, the cell pellet was then fixed in 70% ethanol at 4°C for 15 minutes, under-layered with 1 ml of ice-cold calf serum, and centrifuged for 3 minutes at 300 g. The supernatant was carefully aspirated and the wall of the tube was wiped out with a cotton swab to remove any attached debris. The cell pellet was washed in 2 ml of PBS and incubated in 125 μl of ribonuclease solution (Sigma Chemical Co., St. Louis, MO, USA) at 37°C for 15 minutes. After the water bath incubation, 125 μl of the propidium iodide solution (Sigma Chemical Co., St. Louis, MO, USA) was added and the sample was allowed to stand at room temperature for at least 30 minutes before applying to FC analysis. Over 10,000 cells were included in one test sample.

DNA content measurement was performed on the FACS Calibur flow cytometer (Becton Dickinson, CA, USA), which was equipped with an argon laser set at an emission wavelength of 488 nm and daily calibrated with marked chicken erythrocytes (The QC-particles KIT, Becton Dickinson, CA, USA). The computer program ModFitLT software version 2.0, provided by the manufacturer, was used to perform DNA ploidy analysis.

For analysis and interpretation of DNA ploidy histograms, we followed the conventional standards described previously [9]. Information on the number of cells included in the histogram, number of cells in the peaks, DNA index (DI) of the cells in a peak, tissue aggregates and the coefficient of variation (CV) were provided in the computer read-out. The histograms with single diploid peaks with DI between 0.9 and 1.1 were diagnosed as DNA diploidy, those with an additional second peak (DI between 1.1 and 1.9, or > 2.1), were diagnosed as aneuploid, and those with a second peak with DI between 1.9 and 2.1 and containing > 6% cells were diagnosed as tetraploid [9,23].

### Image cytometry on sections (ICS)

ICS was performed with the ACIS (Clarient Inc., San Juan Capistrano, CA, USA). The system was calibrated daily using a standard procedure to ensure proper focus, black and white level balance for each microscope objective and linear camera outputs. The protocol for DNA content analysis on ICS using the ACIS was the same as that described previously [18,20]. Briefly, a 7 μm-thick section ( ± less than 0.5%) was cut on the Leica RM2155 microtome from the tissue block and stained using the Feulgen Blue Stain kit (Clarient Inc., San Juan Capistrano CA, USA), according to the manufacturer's instructions. The 7 μm-thick section provides information on interphase nuclei [19]. Uniformly stained tissue sections were automatically scanned into the ACIS and digital images were stored using the ACIS-Modifit software. The area of interest was identified microscopically on the H&E stained sections by the pathologist (Q.H.), and the corresponding area on the adjacent Feulgen-stained section was identified by ACIS operators (C.Y. and X.Z.), and visualized under 40× magnification. ACIS uses a set of image processing algorithms known as Watershed Segmentation to exclude touching nuclei. In these algorithms, only the separated nuclei are chosen automatically by the ACIS or manually chosen by the operator; nuclei that touch each other are recognized by the ACIS through their size and other morphometric parameters and are separated by insertion of a single pixel-wide boundary at the point of contact. Overlapping nuclei, nuclear debris and other artifacts that escaped auto-detection and removal by the system were deleted by the operator and not used for analysis. The digital images of nuclei of interest were stored individually and converted into a series of pixels that were quantified as the integrated optical density (IOD) value, representing the DNA content and morphological features of the cells, such as size, shape, contour, granularity and chromatin texture of the nucleus.

Fifty non-epithelial cells on the same section, including endothelial cells, macrophages, fibroblasts, and large lymphocytes, were served as internal reference diploid cells, and the mean IOD value of these cells was assigned a DI value of 1.0, corresponding to 2C or 2N (C for copy number of the chromosomes and N for number of copies of the chromosomes) described in the literature [24]. Two hundred epithelial cells in the target area were then selected and the DI of each cell was calculated with the reference to IOD of control cells. The DNA ploidy histogram of control and target cells was then automatically generated by the system, and the IOD range of each column in the histogram was set as 0.3. The digital image of each selected cell and the corresponding DI value were stored and available for later review if necessary.

The DNA histogram obtained by ICS on ACIS was analyzed as described earlier [20]. Cases with peak DI between 0.9 and 1.1 were diagnosed as diploid and with peak DI > 1.1 as aneuploid. Aneuploidy was further divided into mild (DI: 1.1–1.3), moderate (DI: 1.3–1.8) and severe (DI > 1.8). Peaks with DI vales between 1.8 and 2.2 were also classified as tetraploid that is a specific form of aneuploidy.

Heterogeneity index (HI) was defined as the number of clusters of cells with IOD at 0.3 intervals in the histogram [20]. Histograms with cells with DI > G2 values were identified. Since the G2 value depends on DI of the primary G0/G1 peak, cells with > 5N represent G2 exceeding cells in the histograms with the peak DI of < 1.25; and cells with > 9N represent G2 exceeding cells for the peak DI between 1.25 and 2.25.

### Statistical analysis

All values were described as Mean ± SD. Comparison of mean values of cytometric analysis between groups was carried out with the one-way ANOVA test. The Chi-square test was used to compare the relative portions of cases with positive or negative diagnosis by two methods. Correlation analyses were performed using the SPSS^® ^for Windows statistical package (SPSS Inc., Version 12.0, and Chicago, IL, USA). The two-tailed *P *value of 0.05 or less was considered significant.

## Results

### DNA flow cytometry (FC)

**Figure 1:**
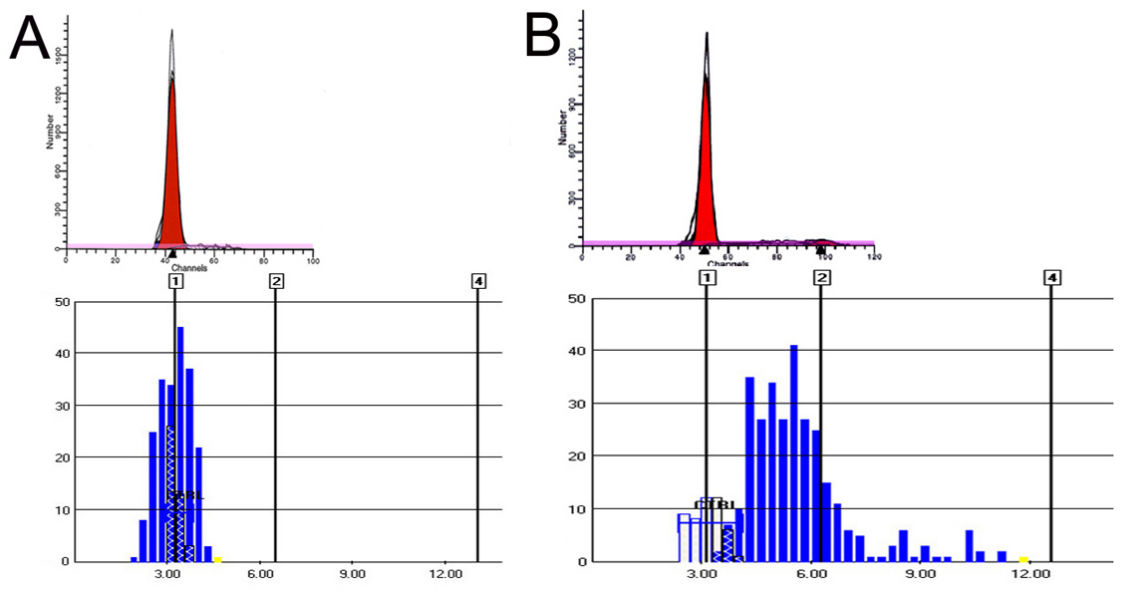
****Concordance of DNA diploidy on standard FC and ICS in a control but not in BAC**.*A***: Normal gastric mucosa shows a diploid DNA histogram on standard FC as well as on ICS. Note that the diploid peak is sharp and narrow (CV = 4.18%) on standard FC and on ICS. There is no increased scatter of cells outside the peak. ***B***: BAC shows a DNA diploid histogram on standard FC but a DNA aneuploid histogram on ICS. The top panel shows a DNA diploid (2N) peak on flow FC. The bottom panel on ICS shows no diploid peak but a wide and short aneuploid peak with peak DI of 1.76, as well as cells with different DI values but no cells with DNA content > 9N. (X-axis shows IOD values and the vertical lines mark DI values).

**Figure 2:**
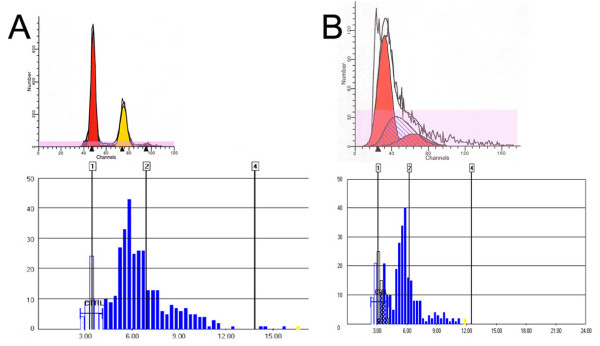
**Concordance of DNA aneuploidy/tetraploidy on standard FC and ICS in BAC.*A***: BAC shows a DNA aneuploid histogram on FC as well as on ICS. The top panel shows a DNA diploid (2N) peak and an aneuploid peak on FC. The aneuploid peak on standard FC had a DI value of 1.6. The bottom panel shows that on ICS, no diploid peak was seen but a broad aneuploid peak with DI value of 1.69, and a large scatter of cells and some cells with DNA content > 9N (see marker for DI of 4). ***B***: BAC shows a DNA tetraploid histogram on standard FC and ICS. The top panel shows a DNA diploid (2N) peak and a tetraploid peak on standard FC. Note that the diploid peak has a large CV (21.41%), representing a diploid with an overlapping paradiploid peak. The bottom panel shows, on ICS, a small diploid peak but a large broad tetraploid peak with DI value of 1.87. Also note a large scatter of cells with different DNA contents but no cells with DNA content > 9N (X-axis shows IOD values and the vertical lines mark DI values).

A total of 10925 ± 1 054 cells were analyzed by FC in 10 mucosal controls. They showed only a diploid peak containing 85 ± 7.9% (range = 71.65% – 93.15%) of cells, with CV of 5.5 ± 1.4% (range = 3.4% – 7.5%). In BACs, standard FC revealed three different DNA ploidy pattern, namely, diploid, aneuploid, and tetraploid (Figures 1 and 2). The DNA diploid pattern was seen in 12/42 (29%) of BAC samples. They included 6,096 ± 1,518 cells and the diploid peaks contained 84.6 ± 8.0% (range = 68% – 94%) cells, with CV of 6.8 ± 2.7% (range = 3.45% – 11.13%).

Thirty out of 42 (71%) BAC samples showed a second aneuploid or tetraploid peak, in addition to the diploid peak. The second peak had DI values between 1.18 and 2.36. Twenty four out of 42 (57%) of BAC samples were diagnosed aneuploid. They had a second aneuploid peak with DI values between 1.18 and 1.9 or between 2.10 and 2.36. These histograms included 5,870 ± 1,179 cells; the aneuploid peak contained 18.0 ± 15.8% (range = 2.3% – 50.6%) of the total cells and had the CV of 5.5 ± 2.4% (range = 2.87% – 13.5%). Six out of 42 (14%) of BAC were diagnosed tetraploid. They had a second tetraploid peak containing > 6% cells with DI values between 1.9 and 2.1. These histograms included 5,716 ± 1,357 cells and the tetraploid region included 8.6 ± 2.7% of the total cells (range = 6.0% – 12.8%). Overall, standard FC diagnosed diploidy in 29%, aneuploidy in 57%, and tetraploidy in 14% of BAC cases. The cell numbers included in the BAC ploidy histograms showing diploidy, aneuploidy or tetraploidy were not statistically different (*p *> 0.8).

Reproducibility of the diagnosis of DNA aneuploidy in different regions of the same BAC tumor by standard FC was investigated in 12 cases. The study showed that the diagnosis of aneuploidy in different regions of the same tumor was reproducible in 6/12 (50%) cases.

### DNA ICS using ACIS

All control normal gastrointestinal mucosal samples showed diploid peaks with DI values between 0.9 and 1.1. The HI was 11.3 ± 1.1, and there were no cells with DI values greater than G2 or > 5N.

In contrast, the main peaks in all BAC samples were either moderately (DI: 1.3–1.8) or severely (peak DI > 1.8) aneuploid. Sometimes, multiple overlapping peaks were noted. HI was markedly elevated with a value of 32.4 ± 8.5. The difference in HI between the control and BAC cells was statistically highly significant (p < 0.001). Cells with DI exceeding G2 was represented by > 5N for the G0/G1 peaks with DI < 1.25 and > 9N for those with peak DI < 2.5. None of the BAC cases had peak DI < 1.25 but 37 cases had peak DI between 1.25 and < 2.25; 19/37 (51%) of these histograms showed cells with DI > 9N.

**Figure 3:**
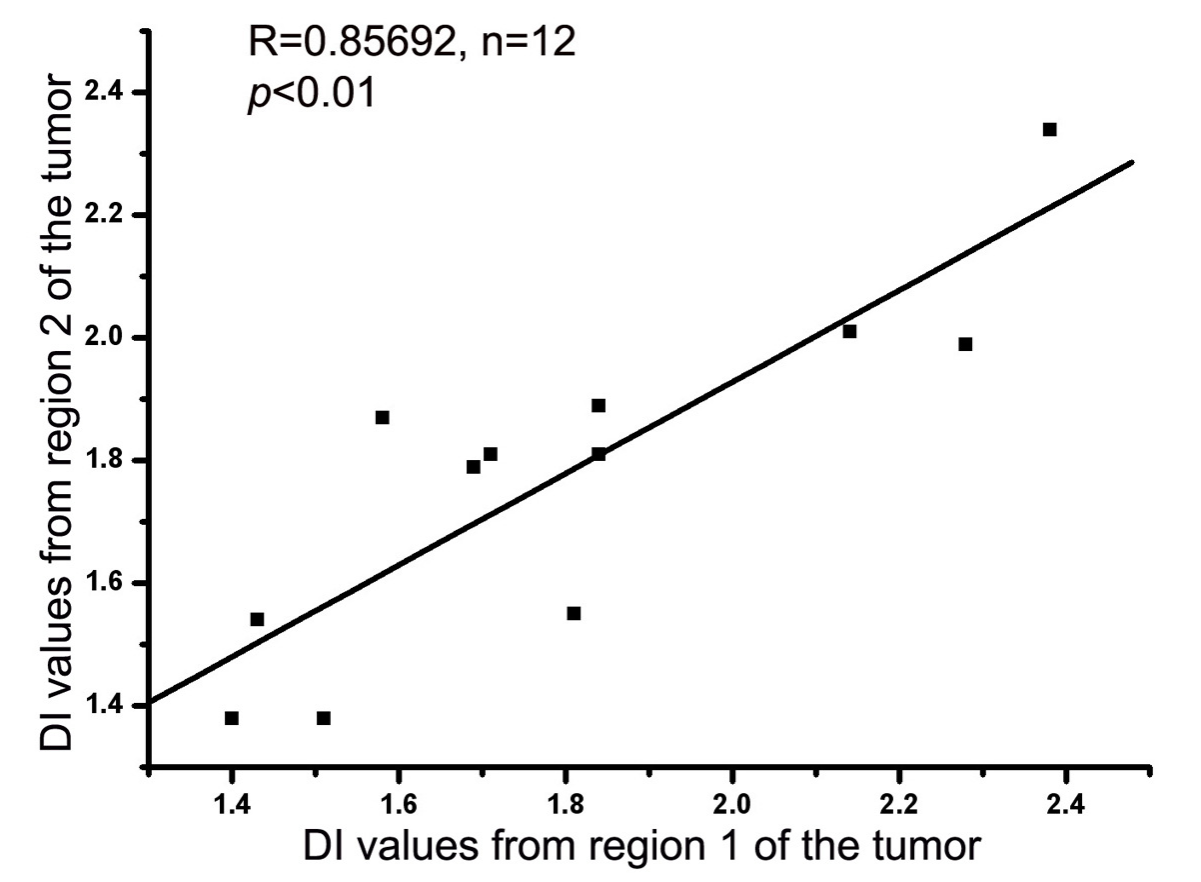
**Correlation of DI values on ICS between two different tissue blocks from the same BAC.** Note that there is a good correlation between the DI values of the aneuploid peaks in tissues from two sites of the same BAC.

There was no intra-tumor heterogeneity in aneuploidy diagnosis by ICS as all BAC samples from different regions of the same tumor were aneuploid. As shown in Figure 3, there was an excellent correlation between DI values of two different regions of the same tumor (R = 0.857, *p *< 0.01).

### Comparison of DNA ploidy profiles between FC and ICS

As shown in Figures 1, 2 and Table 1, normal gastrointestinal mucosa produced normal diploid DNA histograms on both standard FC and ICS. However, the CV of diploid peaks by ICS was significantly larger than that by standard FC (15.8 ± 2.8% vs. 5.5 ± 1.4%; *p *< 0.05).

**Table 1:** Comparison of DNA ploidy status in normal mucosa and BACs generated with FC and ICS

DNA ploidy status	Normal mucosa		BAC	
	
	FC	ICS	FC	ICS
Cases with Diploidy	100%	100%	29%	0
Cases with aneuploidy/tetraploidy	0	0	71%	100%
Heterogeneity Index (mean ± SD)	--	11.3 ± 1.1	--	32.4 ± 8.5
Cases with elevated G2 exceeding fraction (9N)*	--	0	--	51%
Total	10	10	42	42

*Elevated > G2 exceeding fraction include cases showing cells with DI > 9N in the histograms with peak DI between > 1.25 and < 2.25 that was seen in 19 out of 37 cases.

Standard FC diagnosed DNA diploidy in 12/42 (29%) and aneuploidy/tetraploidy in 30/42 (71%) BAC. In contrast, all diploid BAC samples by FC were shown to be aneuploid on ICS (*p *< 0.01) and all aneuploid BAC by FC remained aneuploid on ICS. Moreover, the elevated 9N exceeding fraction was seen in 19/37 (49%) of BAC samples, with peak DI between 1.25 and 2.25, by ICS but FC did not detect 9N exceeding cells in any BAC samples.

## Discussion

The results of this study in BACs show that: 1) Standard DNA FC yields high rates of false negative results in diagnosing aneuploidy and produces low fidelity histograms that overlook minor severe aneuploid clones of cells; 2) In contrast, high fidelity DNA ICS eliminates false negative results and produces high fidelity DNA histogram that precisely identifies minor significant clones.

In this study, standard FC diagnosed 29% of BAC as diploid. This rate of diploid BAC is similar to that reported by others, showing that about 30% of BAC were DNA diploid by FC [6,7,25]. In contrast, all BAC cases including those diagnosed as diploid on FC were aneuploid on ICS. It could be argued that cases of BAC found to be diploid by FC are truly diploid and are falsely diagnosed positive for aneuploidy by ICS. However, this is unlikely because ICS did not yield any false positive diagnosis of aneuploidy in normal control cases that were all correctly identified as diploid. Moreover, the finding of DNA aneuploidy in all BAC samples examined by ICS is similar to other previous reports [17,20]. These observations are also supported by the results of comparative genomic hybridization and karyotyping studies that show major chromosomal alterations in almost all BAC cases [26,27]. These observations suggest that all BAC tumors are aneuploid.

The false negative diagnosis of DNA aneuploidy in BAC on FC in this study may be due to technical limitations, such as inclusion of fewer than 10 000 cells in a DNA histogram. However, this is unlikely. Although greater than 10 000 cells are required for analysis of the S-phase, a smaller number of cells are still sufficient for identification of aneuploid peaks [9]. Moreover, in our study, the number of tumor cells included for analysis in BAC with diploid DNA histograms was not different from that in BAC showing DNA aneuploid or tetraploid histograms.

The main reason for the false negative aneuploidy result in BAC by standard FC appears to be the inclusion of disproportionately large number of non-cancerous diploid cells in the samples. This view is supported by the studies of Rickes and colleagues [10] who reported that many cases of esophageal squamous cell carcinoma or adenocarcinoma that were diploid on standard FC were aneuploid by the double-labeled FC in which esophageal cancer cells were labeled with cytokeratin-19 for squamous cell carcinoma and cytokeratin-18 for adenocarcinoma [10]. Similarly, all cases of BAC were found to be aneuploid by image cytometry that performs DNA content analysis on identified abnormal cells [17,20]. These results are also similar to those on breast cancer, in which many breast carcinomas diagnosed as diploid by FC were aneuploid by ICS [28,29].

Because of the high false negative rate, DNA aneuploidy diagnosis by standard FC was not reproducible in different regions of the same BAC, as shown in this study, resulting in false diagnosis of tumor heterogeneity. Similar findings have been reported in breast carcinoma [30,31]. The lack of reproducibility of DNA aneuploidy diagnosis by standard FC was previously thought to be due to intra-tumor heterogeneity and studies on multiple samples have been recommended for proper evaluations of DNA aneuploidy in BAC [30,31]. However, the present study shows that by ICS all regions of a BAC were aneuploid, which supports the notion that tissue sampling may affect diagnosis of DNA ploidy by standard FC but not by ICS. These results are similar to those reported in breast carcinoma, suggesting that the reported intra-tumor DNA ploidy heterogeneity was due to differences in the techniques used for DNA ploidy analysis [30].

DNA FC technique uses dispersed cell from the whole tissue. Studies using dispersed cells are subject to artifact distortions related to the quality of cell dispersal that determines the amount of cellular debris and aggregates, which affect the DNA histograms[9]. Moreover, unselected dispersed cells from tissues represent mixed populations of abnormal cells (cells of interest) and non-epithelial cells. The proportion of abnormal epithelial and non-epithelial cells varies with the source (e.g., superficial mucosal biopsy vs. deep surgical section) and the cellular composition of the tissue [10]. Therefore, studies on unselected dispersed cells are highly dependent on tissue selection. The instruments and analytical methods used for DNA histogram construction and the criteria for diagnosis of aneuploidy may also affect the results. Inter-laboratory consistency and reproducibility of the FC results have been improved over the years with the development of standardized cell dispersion methods, quality control measures, and improved analytical statistical packages [4,32].

The DNA histograms by FC are biased towards major peaks at the cost of minor peaks and show dominant diploid peaks in all cancer cases [15]. The dominant diploid peaks in FC of BAC may represent contaminated non-cancerous cells in the sample. Because of the bias towards the large peaks, minor but significant peaks and individual severe aneuploid cells are ignored and masked by the background noise in the FC-histograms. Therefore, standard FC does not faithfully detect DNA content heterogeneity or scattering of cells in the histogram.

In contrast to FC, high fidelity ICS identified all BAC tissues as aneuploid and the histograms showed lack of diploid peaks as also reported previously [10,17]. The main reason for these findings may be related to the fact that in ICS selected tumor cells, primarily, are included for DNA content analysis. Moreover, these histograms also identify each cell including the minor aneuploid clones in the histogram and provide an excellent estimate cellular DNA content heterogeneity and G2 exceeding fractions [20]. Another advantage of this technique is that the tissue sections tested as well as the image of each cell and its estimated DNA content are stored in the ACIS system for future verification when needed.

However, ICS has some potential limitations in nuclear DNA content determination related to thickness of the sections [33]. Thick sections (~15 μm) may present many overlapping nuclei of adjacent cells resulting in overestimation of DNA content [19], whereas, thin sections (4 μm – 5 μm) lead to truncated nuclei resulting in underestimation of the DNA content. Such thin sections require the use of correction factors to compensate for the underestimate [16]. Thus, estimation of nuclear DNA values on sections may be sufficiently precise only if the thickness of each individual section is known and the nuclear IOD-values have mathematically been corrected for the error caused by different thickness of sections [33]. This technical challenge has been mitigated to some extent by obtaining uniform sections of 7 μm to 8 μm sections that yield optimal results for study of interphase nuclei [11,19]. The ACIS uses a set of image processing algorithms known as Watershed Segmentation to exclude touching nuclei [18,20]. Overlapping nuclei, nuclear debris, and other artifacts that escape auto-detection and removal by the ACIS are edited out by the operator.

Another potential problem in ICS is operator bias in selecting 'cells of interest'. Selection of abnormal cancer cells may lead to a positive aneuploidy result whereas inclusion of normal epithelial cells may produce a false negative aneuploidy result. In this study, the operator bias was avoided by a two-step process in which the operator was blinded to the histological diagnosis. The operator selected all qualified artifact-free cells in the area of interest that was marked by the pathologist on the adjacent H&E stained section.

In this study DNA histograms obtained by FC and ICS were compared in normal mucosa and BAC because their histological diagnosis is not subject to observer variability. These studies show that both FC and ICS faithfully yield diploid DNA histograms in normal gastrointestinal mucosa, but ICS identifies aneuploidy in BAC samples that are diagnosed as diploid by FC. The high false negative rate of diagnosis of aneuploidy in BAC suggests that FC may also underestimate aneuploid cases of Barrett's dysplasia. Therefore, usefulness of DNA ploidy status by standard FC in confirmation of histological diagnosis of dysplasia or as a clinical biomarker for neoplastic progression in BAC may be limited and require reevaluation [3,4].

Studies using high fidelity ICS have shown that the frequency and severity of aneuploidy by ICS progressively increases with increasing histological grades of dysplasia [20]. Moreover, some of these cases show DNA histograms with greatly increased DNA content heterogeneity and increased number of cells with DNA content greater than their G2 phase, such as increased > 5N cells in the histograms with DI of the peak < 1.25, and cells > 9N in the histograms with DI of peak < 2.5. Increased DNA heterogeneity and elevated G2 exceeding fractions may be important indicators for genomic instability and neoplastic progression. ICS also has the advantage of use in clinical practice as it needs only a small tissue sample and creates a permanent record for independent verification. All these features make ICS a highly desirable tool for DNA ploidy determination in neoplastic conditions. Further studies are warranted to examine the usefulness of DNA content analysis by ICS in discriminating between benign and malignant lesions, identifying patients at high risk for developing dysplasia or carcinoma, monitoring neoplastic progression, and predicting outcomes and responses to treatment of certain types of gastrointestinal cancer including BAC. In this study we only compared FC and ICS. Further studies are needed that directly compare the results of ICS with image cytometry on dispersed cell.

## Conclusion

This study shows that high fidelity ICS is more sensitive and specific than standard FC for detection of DNA aneuploidy in BAC. The high false negative diagnostic rate of DNA aneuploidy in the adenocarcinoma on FC may result from factors such as sampling errors and dilution effects by non-neoplastic cells. High fidelity ICS also provides additional information relevant to neoplastic progression such as HI and G2 exceeding rates. Moreover, this method uses small amounts of biopsy tissues and can be easily extended to tissue samples submitted for conventional histopathological evaluation. A permanent record of the data that can be easily recalled for reexamination, if needed, further adds to usefulness of this technique in clinical practice.

## Abbreviations

FC: flow cytometry; BAC: Barrett's adenocarcinoma; ICS: Image cytometry on tissue sections; ACIS: Automated Cellular Imaging System; VA: Veterans Affairs; H&E: hematoxylin and eosin; PBS: phosphate buffered saline; BSA: bovine serum albumin; CV: coefficient of variation; DI: DNA index; IOD: integrated optical density; HI: Heterogeneity index

## Competing interests

The authors declare that they have no competing interests.

## Authors' contributions

CY and XZ carried out the ACIS analysis and flow cytometry studies. QH participated in the design of the study and was in charge of the pathological study. RKG conceived of the study, and participated in its design and coordination and helped to draft the manuscript. All authors read and approved the final manuscript.

## Pre-publication history

The pre-publication history for this paper can be accessed here:



## References

[B1] Dressler LG, Bartow SA (1989). DNA flow cytometry in solid tumors: practical aspects and clinical applications. Semin Diagn Pathol.

[B2] Ross J (1996). DNA ploidy and cell cycle analysis in pathology..

[B3] Grabsch H, Kerr D, Quirke P (2004). Is there a case for routine clinical application of ploidy measurements in gastrointestinal tumours?. Histopathology.

[B4] Reid BJ, Blount PL, Rabinovitch PS (2003). Biomarkers in Barrett's esophagus. Gastrointest Endosc Clin N Am.

[B5] Teodori L, Gohde W, Persiani M, Ferrario F, Tirindelli Danesi D, Scarpignato C, Di Tondo U, Alo P, Capurso L (1998). DNA/protein flow cytometry as a predictive marker of malignancy in dysplasia-free Barrett's esophagus: thirteen-year follow-up study on a cohort of patients. Cytometry.

[B6] Flejou JF, Doublet B, Potet F, Metayer J, Hemet J (1990). DNA ploidy in adenocarcinoma of Barrett's esophagus. Ann Pathol.

[B7] Reid BJ, Sanchez CA, Blount PL, Levine DS (1993). Barrett's esophagus: cell cycle abnormalities in advancing stages of neoplastic progression. Gastroenterology.

[B8] Robaszkiewicz M, Hardy E, Volant A, Nousbaum JB, Cauvin JM, Calament G, Robert FX, Saleun JP, Gouerou H (1991). [Flow cytometric analysis of cellular DNA content in Barret's esophagus. A study of 66 cases]. Gastroenterol Clin Biol.

[B9] Shankey TV, Rabinovitch PS, Bagwell B, Bauer KD, Duque RE, Hedley DW, Mayall BH, Wheeless L, Cox C (1993). Guidelines for implementation of clinical DNA cytometry. International Society for Analytical Cytology. Cytometry.

[B10] Rickes S, Hauptmann S, Flath B, Abbenseth R, Zwiebel FM, Possinger K (2003). Development of a flow cytometric method to determine DNA ploidy of oesophageal cancer cells obtained by forceps biopsy samples during oesophago-gastro-duodenoscopy. Onkologie.

[B11] Danque PO, Chen HB, Patil J, Jagirdar J, Orsatti G, Paronetto F (1993). Image analysis versus flow cytometry for DNA ploidy quantitation of solid tumors: a comparison of six methods of sample preparation. Mod Pathol.

[B12] Kronenwett U, Huwendiek S, Ostring C, Portwood N, Roblick UJ, Pawitan Y, Alaiya A, Sennerstam R, Zetterberg A, Auer G (2004). Improved grading of breast adenocarcinomas based on genomic instability. Cancer Res.

[B13] Lorenzato M, Abboud P, Masure M, Bouttens D, Visseaux-Coletto B, Quereux C, Adnet JJ (2000). Image cytometry detection of breast cancer cells with > 5C DNA content and minor DNA stemlines. Anal Quant Cytol Histol.

[B14] Yildirim-Assaf S, Coumbos A, Hopfenmuller W, Foss HD, Stein H, Kuhn W (2007). The prognostic significance of determining DNA content in breast cancer by DNA image cytometry: the role of high grade aneuploidy in node negative breast cancer. J Clin Pathol.

[B15] Auer G, Askensten U, Ahrens O (1989). Cytophotometry. Hum Pathol.

[B16] Bacus JW, Bacus JV (1994). A method of correcting DNA ploidy measurements in tissue sections. Mod Pathol.

[B17] Fang M, Lew E, Klein M, Sebo T, Su Y, Goyal R (2004). DNA abnormalities as marker of risk for progression of Barrett's esophagus to adenocarcinoma: image cytometric DNA analysis in formalin-fixed tissues. Am J Gastroenterol.

[B18] Huang Q, Yu C, Klein M, Fang J, Goyal RK (2005). DNA index determination with Automated Cellular Imaging System (ACIS) in Barrett's esophagus: comparison with CAS 200. BMC Clin Pathol.

[B19] Steinbeck RG, Auer GU, Zetterberg AD (1999). Reliability and significance of DNA measurements in interphase nuclei and division figures in histological sections. Eur J Cancer.

[B20] Yu C, Zhang X, Huang Q, Klein M, Goyal RK (2007). High-fidelity DNA histograms in neoplastic progression in Barrett's esophagus. Lab Invest.

[B21] Joensuu H, Alanen KA, Klemi PJ, Aine R (1990). Evidence for false aneuploid peaks in flow cytometric analysis of paraffin-embedded tissue. Cytometry.

[B22] Haggitt RC (1994). Barrett's esophagus, dysplasia, and adenocarcinoma. Hum Pathol.

[B23] Rabinovitch PS, Longton G, Blount PL, Levine DS, Reid BJ (2001). Predictors of progression in Barrett's esophagus III: baseline flow cytometric variables. Am J Gastroenterol.

[B24] Haroske G, Giroud F, Reith A, Bocking A (1998). 1997 ESACP consensus report on diagnostic DNA image cytometry. Part I: basic considerations and recommendations for preparation, measurement and interpretation. European Society for Analytical Cellular Pathology. Anal Cell Pathol.

[B25] Robaszkiewicz M, Reid BJ, Volant A, Cauvin JM, Rabinovitch PS, Gouerou H (1991). Flow-cytometric DNA content analysis of esophageal squamous cell carcinomas. Gastroenterology.

[B26] Riegman PH, Vissers KJ, Alers JC, Geelen E, Hop WC, Tilanus HW, van Dekken H (2001). Genomic alterations in malignant transformation of Barrett's esophagus. Cancer Res.

[B27] Walch AK, Zitzelsberger HF, Bruch J, Keller G, Angermeier D, Aubele MM, Mueller J, Stein H, Braselmann H, Siewert JR, Hofler H, Werner M (2000). Chromosomal imbalances in Barrett's adenocarcinoma and the metaplasia-dysplasia-carcinoma sequence. Am J Pathol.

[B28] Alanen KA, Lintu M, Joensuu H (1998). Image cytometry of breast carcinomas that are DNA diploid by flow cytometry: time to revise the concept of DNA diploidy?. Anal Quant Cytol Histol.

[B29] Susnik B, Poulin N, Phillips D, LeRiche J, Palcic B (1995). Comparison of DNA measurement performed by flow and image cytometry of embedded breast tissue sections. Anal Quant Cytol Histol.

[B30] Askensten UG, von Rosen AK, Nilsson RS, Auer GU (1989). Intratumoral variations in DNA distribution patterns in mammary adenocarcinomas. Cytometry.

[B31] Bergers E, van Diest PJ, Baak JP (1996). Tumour heterogeneity of DNA cell cycle variables in breast cancer measured by flow cytometry. J Clin Pathol.

[B32] Bergers E, Montironi R, van Diest PJ, Prete E, Baak JP (1996). Interlaboratory reproducibility of semiautomated cell cycle analysis of flow cytometry DNA-histograms obtained from fresh material of 1,295 breast cancer cases. Hum Pathol.

[B33] Mairinger T, Gschwendtner A (1996). Comparison of different mathematical algorithms to correct DNA-histograms obtained by measurements on thin liver tissue sections. Anal Cell Pathol.

